# A Missing Traffic Data Imputation Method Based on a Diffusion Convolutional Neural Network–Generative Adversarial Network

**DOI:** 10.3390/s23239601

**Published:** 2023-12-04

**Authors:** Chenchen Zhang, Lei Zhou, Xuemei Xiao, Dongwei Xu

**Affiliations:** 1 School of Mechanical and Automotive Engineering, Xiamen University of Technology, Xiamen 361024, China; zhangchenchen@xmut.edu.cn (C.Z.); xiaoxuemei@xmut.edu.cn (X.X.); 2 Institute of Cyberspace Security, Zhejiang University of Technology, Hangzhou 310023, China; 201806060423@zjut.edu.cn; 3 College of Information Engineering, Zhejiang University of Technology, Hangzhou 310023, China

**Keywords:** graph embedding, deepwalk, generative adversarial network, diffusion convolutional neural networks, data imputation

## Abstract

Traffic state data are key to the proper operation of intelligent transportation systems (ITS). However, traffic detectors often receive environmental factors that cause missing values in the collected traffic state data. Therefore, aiming at the above problem, a method for imputing missing traffic state data based on a Diffusion Convolutional Neural Network–Generative Adversarial Network (DCNN-GAN) is proposed in this paper. The proposed method uses a graph embedding algorithm to construct a road network structure based on spatial correlation instead of the original road network structure; through the use of a GAN for confrontation training, it is possible to generate missing traffic state data based on the known data of the road network. In the generator, the spatiotemporal features of the reconstructed road network are extracted by the DCNN to realize the imputation. Two real traffic datasets were used to verify the effectiveness of this method, with the results of the proposed model proving better than those of the other models used for comparison.

## 1. Introduction

Today, improvements in the economic level and the rise in per-capita vehicle ownership have improved travel efficiency while further accelerating the pace of social development. This increasing vehicle ownership presents great challenges in the use of road network resources, such as the rising incidence of traffic congestion and accidents in urban areas. Therefore, the macro-level control of traffic resources is particularly important. In daily life, travel modes and the macro-level control of traffic management departments depend on data gathered through traffic detection systems. These datasets are utilized across various sectors, including predicting traffic flows and their distributions. Due to both external factors (rainstorm, power failure, signal disturbance, etc.) and internal factors (internal failure, need for repair, etc.) the detectors arranged in the traffic network may not always be able to collect data at all times [1]. For example, in California’s performance measurement system (PEMs), the data missing rate sampled every day is 15% [2], while the data missing rate sampled by the Texas Transportation Research Institute is between 16% and 93% [3]. However, many subsequent traffic control and road network resource allocation tasks are based on the assumption of complete and accurate data. Timely provision of complete data is an important guarantee for the stable completion of various downstream tasks [4]. Therefore, it is of practical significance to utilize traffic state data to deduce and generate the missing data. This process can aid in the completion of subsequent tasks within the ITS, ensuring the accurate management of traffic resources even when road network data are missing.

Three primary categories of missing data exist [5]. When the traffic state data are missing completely at random (MCAR), the deletion probability of all observations is the same, that is, the missing points are a random subset of the full dataset. On the other hand, when data are not missing at random (MNAR), as the name implies, there is a connection between the missing data and some other value. Finally, missing at random (MAR) means that the tendency of data points to be lost is related to other observed data in the dataset, and not to the lost data.

Numerous approaches have been suggested for addressing missing data, and can be broadly categorized into three types [6,7,8]: prediction, interpolation, and statistical learning. Prediction approaches [9,10,11] rely on past data to construct a model to estimate the data. However, these approaches only utilize past data, which can lead to accuracy problems. Interpolation approaches [12,13,14] substitute adjacent or previous data for the missing data. When the daily traffic state data are similar, these approaches can perform satisfactorily’ however, these change with time in the real world. Statistical learning approaches [6,15] typically presuppose a data probability distribution, conduct iterative processes to derive model parameters, and subsequently utilize the available data to deduce the corrupted or missing points. However, these approaches may generate inaccurate imputation results when the traffic state data do not adhere to a particular distribution.

Tp address the existing problems with the above approaches, we propose a method for enhancing the accuracy and stability of imputation results. The primary contributions of the proposed method are outlined below:

1. Deepwalk is used to reconstruct the road network and obtain the node set with the highest spatial correlation of each node as the neighborhood of the node reconstructed to improve the temporal and spatial correlation.
2. DCNN is used to capture the dynamic spatial characteristics and consider the forward and reverse traffic flow diffusion to further enhance adaptability and accuracy.
3. During experimental analysis, two real datasets and several classical missing traffic state data imputation models are selected for comparative experiments to verify the accuracy and robustness of the proposed model.

## 2. Related Work

Generation models can use missing data for training in order to understand the distribution of sample data. This approach minimizes the challenge of acquiring sample data and generates data that closely resemble real samples [16]. Therefore, its applications span various domains, including image generation [17], text classification [18], and image conversion [19]. At present, the main generation models are the Generative Adversarial Network (GAN) [20] and Variational Autoencoder (VAE) [21] models.

GANs were first proposed by Goodflow in 2014. A number of subsequent models have been proposed on this basis, such as Deep Convolutional Generative Adversarial Network (DCGAN) [22], Conditional Generative Adversarial Network (CGAN) [23], and Information Maximizing Generative Adversarial Network (InfoGAN) [24]. When contrasted with conventional neural networks, these models excel at feature extraction and offer a robust structure to learn complicated high-dimensional distributions. These methods find extensive applications across diverse fields [25,26,27,28].

A GAN includes a generator *G* and discriminator *D*. The generator extracts a feature distribution and attempts to generate data that approximate the real data. Meanwhile, the discriminator evaluates whether the input data originate from real data. The objective of a GAN is represented by Equation (1):

(1) $$\min\max E_{x∼p_{data}\left(x\right)}\left[\log(D(x))\right]+E_{z∼p_{z}\left(z\right)}\left[\log(1-D(G(z)))\right]$$

where $P_{z}\left(z\right)$ and $P_{data}\left(x\right)$ represent the standard and training data distributions, respectively.

GANs have recently found broad application in the transportation sector. For example, a GAN was applied to predict the distribution of travel times using trajectory data in [29]. Xu applied a GAN to car-following considering missing data [30]. The internal GAN structure based on a graph convolution network was used to generate traffic speed data in [31]. A pattern-sensitive network utilizing a GAN was introduced for traffic flow prediction in [32]. During training of GANs, the generator and discriminator may encounter problems such as non-convergence, unstable training, and vanishing gradients. Arjovsky proposed the WGAN (Wasserstein Generative Adversarial Network) [33] to overcome these issues.

Graph-based analysis tasks have found extensive utilization in real-world scenarios, such as social networks, transportation networks [34,35], multi-agent networks [36], and more. Networks with a graph structure are usually high-dimensional and difficult to deal with; lately, however the dimensionality reduction of graph structures has developed greatly thanks to graph embedding algorithms. Graph embedding maps graph data into low-dimensional vectors to represent the graph topology. This embedding facilitates further work, including node clustering [37], link prediction [38], node classification [39], and network visualization [40]. Classical graph embedding methods include Deepwalk [41], node-to-vector (Node2vec), and structural deep network embedding (SDNE) [42,43,44]. Deepwalk first constructs a random walk sequence through the graph structure relationship to obtain the node’s local context information, then takes the obtained sequence as a sentence in word2vec, and learns the low-dimensional representation vector of the node through skip-gram [45]. Deepwalk can maintain good performance even when the training dataset is sparse. Because Deepwalk uses a random walk to obtain the local node sequence, the nodes passing through the node’s walk path are selected with equal probability. The more adjacent nodes in the graph two points have, the closer the proximity of the nodes embedded in the vector.

## 3. Methodology

**Figure 1:**
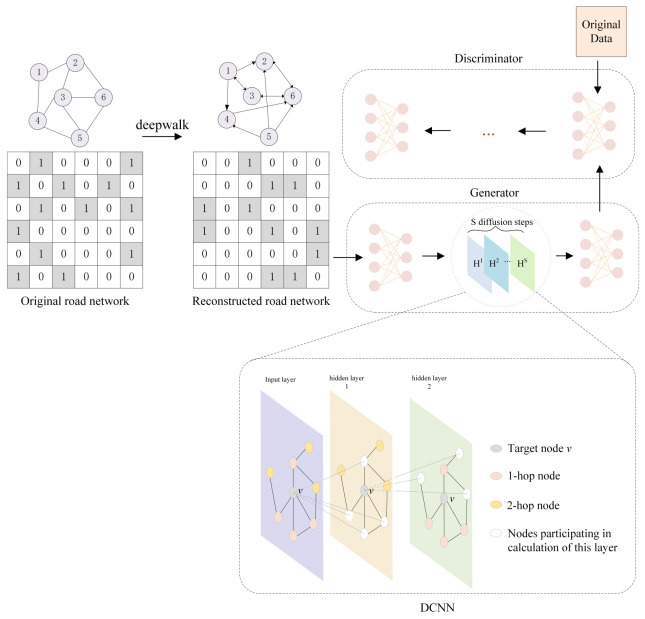
The detailed process of DCNN-GAN.

The overall framework for the imputation method of traffic state data based on DCNN-GAN is shown in Figure 1. First, the traffic state data matrix containing missing data is obtained by masking the original data. Second, the potential representation vector in the road network is obtained using Deepwalk. After finding the detector with the highest relevance, the adjacency relationship of all detectors is reconstructed to obtain a new adjacency matrix. Finally, the spatial features of the graph structure are extracted in the generator part of the GAN using DCNN and the complete data are generated through adversarial training of the GAN to achieve imputation.

### 3.1. Data Definition

The road network is defined as G=(V,E), which includes detector nodes $V=(v_{1},v_{2},\ldots ,v_{N})$ and edges $E={e_{ij}}_{i,j=1}^{N}$, where $N=\left|V\right|$ represents the total count of detectors. The connection relationship between the detectors is obtained from the adjacency matrix $A\in R^{N\times N}$. If a link exists between detector *i* and detector *j*, then $e_{ij}=1$ and $A_{ij}=1$; otherwise, $e_{ij}=0$ and $A_{ij}=0$. The traffic state data are expressed as $x_{i}=\left(x_{i1},x_{i2},\ldots ,x_{iT}\right)$, where $x_{it}$ represents the data of detector *i* at time *t* and *T* indicates the duration of data collection.

During data preprocessing, the traffic state data are divided according to *H* as the minimum time, and the data are divided into T/H samples based on the length *T* of each detector to construct the traffic state dataset $X\in R^{(T/H)*H*N}$. The mask matrix $M\in R^{(T/H)*H*N}$, which shares the same dimensions as *X*, is set comprising values of 0 and 1. The traffic state data matrix containing missing data $\hat{X}=X*M$ is obtained by multiplying the corresponding elements *X* and *M*.

### 3.2. Road Network Reconstruction

The Deepwalk algorithm was proposed in 2014, and was initially applied to graph embedding. This method can express the nodes within a graph as a vector with potential features by acquiring the hidden characteristics of the network. It comprises two primary parts: random walk and skip-gram.

First, a random walk is carried on the original road network G=(V,E). Let $W_{v_{i}}$ represent the path (similarity sequence) obtained from a random walk from node $v_{i}$; then, the similarity sequence of nodes $W_{v_{i}}=\left\{W_{v_{i}}^{1},W_{v_{i}}^{2},\ldots ,W_{v_{i}}^{k}\right\}$ is obtained, where $W_{v_{i}}^{j}$ represents the *j*th node in the random walk process with node $v_{i}$ as the starting point and *k* represents the length of the random walk. The nodes which the random walk selection path pass by can be described as follows. Starting from the initial point of the walk, each subsequent node randomly selects an edge connected to the current node and moves to the next node along the selected edge. For each detector, the random walk is repeated η times to obtain η walk paths, and the length of the path of each random walk is *k*.

Next, using the sequence obtained via random walk, skip-gram is employed to learn the embedded representation of the nodes. First, it is necessary to map node $v_{i}$ to a vector representation through the mapping function $\Phi :v\epsilon V\to R^{\left|V\right|\times d}$. In this way, each node is mapped into a d-dimensional vector in order to learn its potential spatial representation within the road network. We want to maximize the probability that the nodes within the random walk sequence appear in the context window of the missing nodes, that is, $Pr(\Phi \left(v_{i}\right)\left|\left(v_{i-w},\ldots ,v_{i+w}\right)\right)$, where *w* represents the size of the selection window. The optimization objective function is expressed as Equation (2):

(2) $$\min-\log[Pr\left(\Phi \left(v_{i}\right)|\left(v_{i-w},\ldots ,v_{i+w}\right)\right)].$$

The complex formula above can be transformed into Equation (3) by Pearson correlation:

(3) $$Pr\left(\Phi \left(v_{i}\right)|\left(v_{i-w},\ldots ,v_{i+w}\right)\right)=\prod_{j=i-w}^{i+w}Pr(\Phi \left(v_{i}\right)|v_{j}).$$

In the skip-gram algorithm, layered softmax is used to can avoid calculating the softmax of all words when calculating the probability, improving the training efficiency of the model. A Huffman tree is used to replace the mapping from the hidden layer to the output softmax layer, which is constructed according to the frequency of nodes. The count of leaf nodes in the Huffman tree is the count of nodes. In the Huffman tree, the negative class (recorded as 1) represents walking along the left subtree, while the positive class (recorded as 0) represents walking along the right subtree. Using a Huffman tree reduces the amount of calculation and makes higher-frequency nodes easier to find. Let $u_{p}\in \left\{v_{i-w},\cdots ,v_{i+w}\right\}$; then, the path from the root node $\Phi (v_{i})$ to the leaf node $u_{p}$ in a binary tree can be represented by a tree node path $b_{1},b_{2},\cdots ,b_{\left|log|V|\right|}$ and $b_{\left|log|V|\right|}=u_{p}$. Therefore, $Pr\left(u_{p}|\Phi \left(v_{i}\right)\right)$ can be expressed as Equation (4):

(4) $$Pr\left(u_{p}|\Phi \left(v_{i}\right)\right)=\prod_{r=1}^{log|V|}Pr\left(b_{r}|\Phi \left(v_{i}\right)\right).$$

$Pr\left(b_{r}|\Phi \left(v_{i}\right)\right)$ can be modeled by the binary classification problem formed by the allocation of parent node $b_{r}$; the following Equation (5) is obtained, where $\phi \left(b_{r}\right)$ denotes the parent node allocated to tree node $b_{r}$:

(5) $$Pr\left(b_{r}|\Phi \left(v_{i}\right)\right)=\frac{1}{1+e^{-\Phi \left(v_{i}\right)\cdot \phi \left(b_{r}\right)}}.$$

Through the above optimization calculation and the objective optimization function in Equation (3), the vector representation of the potential network structure can be obtained. When applied to the traffic network, each detector can obtain the detector set with the highest relationship to it in space. Therefore, Deepwalk graph embedding is performed for each detector to obtain *m* road segment sets with the highest relationship and the obtained road segment sets are used as the reconstructed adjacency detector of the detector. We let the reconstructed road network adjacency matrix be $\tilde{A}\to R^{\left|V\right|\times \left|V\right|}$ and record the reconstructed road network as $\tilde{G}=\left(\tilde{V},\tilde{E}\right)$.

### 3.3. Generation of Traffic Data

After extracting the representation information of each detector using Deepwalk and reconstructing the adjacency relationship of the detectors, the generation of missing traffic state data is realized via GAN. The framework of the GAN includes a generator *G* and discriminator *D*. The generator *G* learns the data distribution mapped from the input data, and the discriminator *D* tries to determine whether the data are from the real data distribution or the generated data distribution. During model training, the generator *G* and discriminator *D* continuously improve their learning ability; as the generator *G* generates more realistic data distributions, the discriminator *D* further improves its discrimination ability and finally becomes able to generate effective data distributions. Due to a number of problems, traditional GANs are difficult to train; thus, we adopted the Wasserstein GAN (WGAN) for model training.

The generator imputes the complete data by learning the features of the graph structure through a diffusion convolution neural network (DCNN), then uses a sparse adjacency matrix to reduce the computational complexity. The reconstructed graph $\tilde{G}=\left(\tilde{V},\tilde{E}\right)$ is obtained by road network reconstruction. Because the reconstructed adjacency relationship no longer has symmetry, the connection relationship of the nodes in the road network changes from undirected to directed. The DCNN accurately captures traffic flow by modeling traffic flow as a process diffusing across the graph. For each node on different diffusion steps, DCNN obtains the spatial dynamic information of the nodes, that is, the neighbor information in the road network is extracted from 1 to S through the diffusion step. Diffusion step 1 represents the neighbor node of the node, diffusion step 2 represents the second-order neighbor node of the node, and so on. After merging the information extracted by each diffusion step, the spatial aggregation information is obtained, which is extracted by DCNN. In addition, because reverse diffusion can enhance the model’s adaptability to acquire the impact of the traffic flow both upstream and downstream, the diffusion process includes forward diffusion and reverse diffusion.

The complete generator calculation process is shown in Equations (6)–(8):

(6) $$H_{l1}^{G}=\sigma \left(W_{l1}^{G}\cdot X+b_{l1}^{G}\right)$$

(7) $$H_{l2}^{G}=\sigma \left[\left(\sum_{s=1}^{S-1}W_{s,1}^{G}{\left(D_{O}^{-1}\right)}^{s}+W_{s,2}^{G}{\left(D_{I}^{-1}\right)}^{s}\right)H_{l1}^{G}\right]$$

(8) $$\hat{G}=f\left(W_{l3}^{G}\cdot H_{l2}^{G}+b_{l3}^{G}\right)$$

where $W_{l1}^{G}$, $W_{l3}^{G}$, $b_{l1}^{G}$, $b_{l3}^{G}$ respectively represent the weight and bias of the hidden layer, $W_{s,1}^{G}$, $W_{s,2}^{G}$ represent the parameters of the forward and reverse diffusion convolution kernel, *S* is the maximum diffusion step, $D_{O}$ and $D_{I}$ represent the out-degree and in-degree matrix, $D_{O}^{-1}$ and $D_{I}^{-1}$ represent the transfer matrix of the forward and reverse diffusion process, respectively, σ and *f* represent different activation functions, $H_{l1}^{G}$ denotes the features extracted by the first layer, $H_{l2}^{G}$ represents the features extracted by the diffusion convolution layer, and $\hat{G}$ denotes the output of the generator.

The discriminator, which is composed of multi-layer full connection layers, is mainly responsible for distinguishing whether the data is real or generated data using the calculation formula in Equations (9) and (10):

(9) $$H_{l}^{D}=\sigma \left(W_{l}^{D}\cdot H_{l-1}^{D}+b_{l}^{D}\right)$$

(10) $$\hat{D}=W_{o}^{D}\cdot H_{L_{D}}^{D}+b_{o}^{D}$$

where $\hat{D}$ is the output of the discriminator, $L_{D}$ denotes the number of layers of the fully connected layer, $l\in \left[1,L_{D}\right]$, $H_{l-1}^{D}$ is the extracted feature of the former fully connected layer, $H_{0}^{D}$ denotes the initial input (*X* or $\hat{G}$), and σ is the activation function.

After defining the generator and discriminator models, their respective loss functions need to be designed in order to facilitate the effective training of the models. The loss function of the generator is mainly composed of the basic loss function and the data repair loss function, while the loss function of the discriminator is the difference between the real data and the generated data, as shown in Equations (11) and (12):

(11) $$loss_{G}=-\hat{D}\left(\hat{G}\right)+\alpha {\left(\hat{G}-X\right)}^{2},$$

(12) $$loss_{D}=\hat{D}\left(\hat{G}\right)-\hat{D}\left(X\right).$$

## 4. Experimental Results

### 4.1. Experimental Design

**Figure 2:**
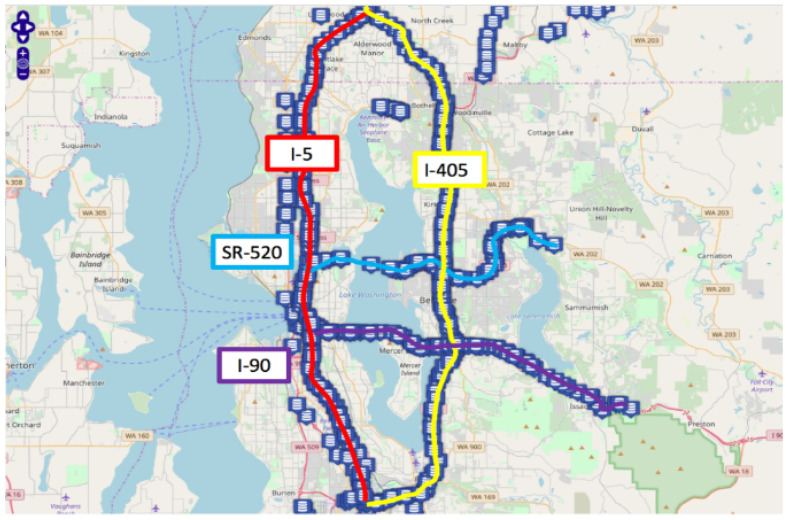
Distribution map of detectors in the Seattle speed dataset.

To ensuring that the experimental results reflected a real scenario, two real-world datasets were selected for experimental verification and analysis. These datasets were the PEMS-BAY flow dataset from California and the Seattle speed data set. The PEMS-BAY dataset consists of data collected from 24 detectors in zone 7 of the PEMS open source website from California. The data sampling period is 5 min, including flow data from 1 May 2014 to 30 June 2014; therefore, each detector contains 17,568 sampling data. The Seattle speed dataset is collected from 323 detectors on four continuously connected highways in Seattle: I-5, I-405, I-90, and sr-520. It records the traffic speed data for the entire year of 2015 at a 5 min sampling interval. Figure 2 shows the detector distribution in the Seattle speed data set.

**Figure 3:**
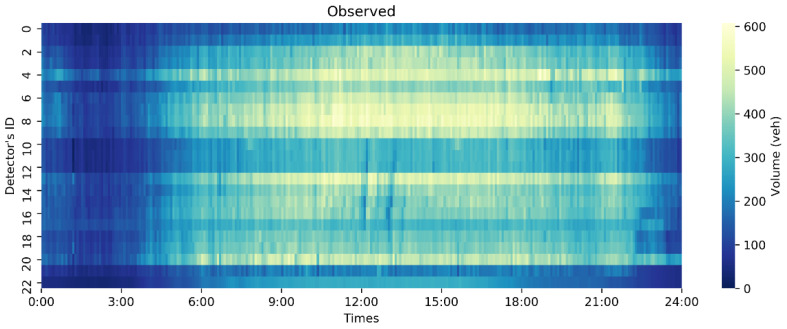
Original complete observed data on 1 May 2014 from the PEMS-BAY dataset.

**Figure 4:**
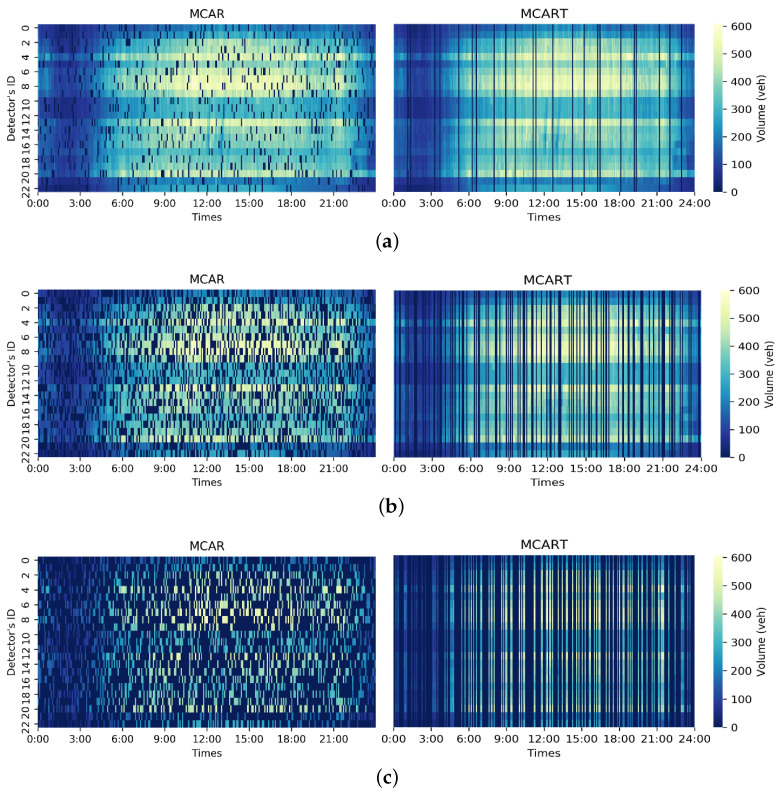
Data distribution of the detectors in the PEMS-BAY dataset under different missing rates and missing modes on 1 May 2014. (**a**) Data distribution with 10% missing rate; (**b**) data distribution with 40% missing rate; (**c**) data distribution with 70% missing rate.

To better simulate a real data loss scenario in the experiment, the data loss modes in the dataset were divided prior to the experiment into missing completely at random (MCAR) and missing completely at random in time (MCART) through mask masking. MCAR refers to data loss that occurs randomly in space and time, while MCART means that all detectors are missing data at a certain time or time interval. Figure 3 shows the real data distribution of the PEMS-BAY dataset on 1 May 2014 and Figure 4 shows the data distribution of complete random deletion under different deletion rates and completely random deletion over time.

In Figure 3 and Figure 4, the abscissa represents the time point, the ordinate represents the detector ID in the road network, and the brightness of the midpoint in the thermal diagram represents the height of the traffic flow, with brighter color indicating greater flow values. The heatmaps in Figure 3 and Figure 4 only represent the traffic flow, and do not include vehicle speed. It can be seen that the peak flow starts after 8:00 and the low peak flow starts after 19:00. In Figure 4, the left side shows the data distribution of traffic flow data under complete random deletion and the right side shows the data distribution of the traffic flow data under complete random deletion in time. As the missing data rate increases, the number of missing grids in the thermodynamic diagram increases gradually. The stability of the model with an increasing deletion rate can be verified. The deletion rates selected in this experiment were 10%, 40%, and 70%.

### 4.2. Model Settings and Evaluation Criteria

The parameters of the DCNN-GAN model are mainly composed of two parts: Deepwalk and GAN. In the Deepwalk module, for PEMS-BAY dataset, the number of highly relevant detectors selected was *m* = 4; for the Seattle dataset, the number of highly relevant detectors selected was *m* = 10. In the Deepwalk module, the fixed parameters were the size of the context window of the node *w* = 5, number of random walks of each node 10, length of random walks *k* = 40, and embedded vector dimension *d* = 64.

In the GAN module, the parameter settings of PEMS-BAY dataset and Seattle dataset were the same. The generator included the full connection layer and DCNN layer. In the DCNN layer, the diffusion convolution step was set as the best parameter in $K\in \left[1,2,3,4\right]$. The number of hidden layer units in the full connection layer of the first layer of the generator was 512, the number of hidden layer units in the DCNN layer was 256, and the number of hidden layer units in the full connection layer of the third layer of the generator was 128. Except for the output layer, the activation function of each layer was the ReLU function; the activation function of the last layer of the generator was the sigmoid function. The discriminator included three fully connected layers, in which the number of hidden layer units in each layer was 512, 216, and 128, respectively. Except for the output layer, the activation function of hidden layers was the ReLU function. Because WGAN was used for countermeasure training, the output layer in the discriminator did not use an activation function.

The dataset was divided into a training set and test set, with the first 80% of the data used to train the model and the other 20% used to test the effectiveness of the model. The training batch size of the model was 512 and the number of training rounds was 20,000. The following model performance evaluation indicators were selected: mean absolute error MAE, root mean square error RMSE, and mean absolute error MAPE. The calculation formulas are shown in Equations (13)–(15):

(13) $$\mathrm{MAE}=\frac{1}{n}\sum_{i=1}^{n}\left|{\hat{y}}_{i}-y_{i}\right|,$$

(14) $$\mathrm{RMSE}=\sqrt{\frac{1}{n}\sum_{i=1}^{n}{\left({\hat{y}}_{i}-y_{i}\right)}^{2}},$$

(15) $$\mathrm{MAPE}=\frac{1}{n}\sum_{i=1}^{n}\left|\frac{{\hat{y}}_{i}-y_{i}}{y_{i}}\right|,$$

where *n* represents the amount of missing data, *y* represents the real data corresponding to the missing data, and ${\hat{y}}_{i}$ represents the repaired data output by the model.

### 4.3. Baseline Methods

The deduction effect of the model can be further verified by selecting an appropriate and effective comparison model. In this experiment, typical algorithms for data repair were selected for comparative experiments. The comparison models were DSAE (denoising stacked auto encoders) [46], Bayesian Gaussian CANDECOMP/PARAFAC (BGCP) [47], and GAN.

The DSAE model had two hidden layers, the number of hidden layer units was 128 and 64, the training batch size of the model was 128, and the number of training rounds was 1000. For the BGCP model, the third-order tensor (number of detectors, days, and time interval) was used as the model input. For the GAN model, the generator and discriminator used three fully connected layers as the hidden layer. Because WGAN was used for training, the output layer in the discriminator did not use an activation function. The training batch size was 512 and the number of training rounds was 20,000.

### 4.4. The Effect of DCNN and Best Diffusion

First, in order to prove the influence of the DCNN layer in DCNN-GAN on the the spatial dependence of the road network and select the best diffusion convolution step S, a parameter determination experiment was carried out on the PEMS-BAY dataset. Several diffusion convolution steps were selected for comparison; the evaluation indexes were RMSE, MAE, MAPE, and training time. In this experiment, the other parameters were the same except for the diffusion convolution step.

**Figure 5:**
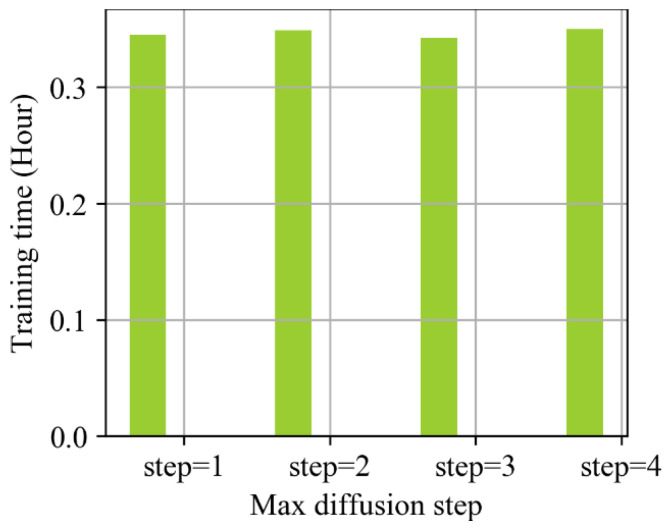
Relationship between model training time and number of diffusion convolution steps.

It can be seen from Table 1 and Figure 5 that when the diffusion convolution step s increases, the repair error of the model shows a downward trend as a whole. When S=2, the repair error of the model is the lowest. When the diffusion convolution step increases further, the error increases. This may be due to the difficulty of transmitting more effective highway data spatial information for nodes with more than a two-order neighborhood in the road network when the diffusion convolution step increases. The training time of the model changes little when the diffusion convolution step size increases. Therefore, a diffusion convolution step size of S=2 was selected as the experimental parameter in the follow-up experiment.

### 4.5. Experiment and Analysis of PEMS-BAY Dataset

**Figure 6:**
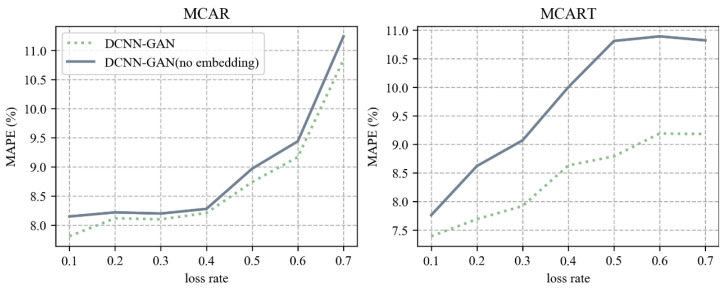
The influence of image embedding on the repair of missing data.

In order to prove the effectiveness of Deepwalk embedding learning in the model, the reconstructed road network obtained by Deepwalk embedding learning and the original road network without Deepwalk embedding learning were compared under different data loss rates. The comparison index was MAPE. The experimental comparison results are shown in Figure 6. In the figure, DCNN-GAN represents the experimental results reconstructed by graph embedding, while DCNN-GAN (no embedding) indicates the experimental results on the original road network. In MCAR and MCART deletion mode, the experimental results of DCNN-GAN are better than those of DCNN-GAN (no embedding).

In MCAR mode, when the deletion rate is less than 40%, the prediction performance of DCNN-GAN and DCNN-GAN (no embedding) remains relatively stable. When the deletion rate exceeds 40%, the repair error gradually increases. This is because in MCAR mode, when the data deletion rate is too high the surrounding detectors are in the state of high data deletion; as a result, too little information is obtained in space and time. DCNN-GAN is always better than DCNN-GAN (no embedding).

In MCART mode, it can be seen that as the deletion increases rate, the repair effect of DCNN-GAN grows significantly better than DCNN-GAN (no embedding). As the data deletion rate increases, the repair error of DCNN-GAN gradually tends towards stability, because the model reconstructs the adjacency matrix according to spatial correlation. In MCART mode, when the time period of the missing data gradually increases, the model is able to maintain its repair performance through spatial dependence, showing good robustness.

Table 2 compares the repair error results of DCNN-GAN and other models in MCAR and MCART modes. It can be seen that the prediction performance of DCNN-GAN in these two modes always remains optimal with the increasing data deletion rate. In MCAR deletion mode, the average MAPE of DCNN-GAN with a deletion ratio from 10% to 70% decreases by about 24.59% compared with DSAE, 5.25% compared with GAN, and 27.89% compared with BGCP. In MCART deletion mode, the average MAPE of DCNN-GAN with a deletion rate from 10% to 70% is about 25.02% lower than that of DSAE, 13.95% lower than GAN, and 13.42% lower than BGCP. Under the different data deletion rates of the two data deletion models, the prediction error of DCNN-GAN is always lower than the other models, and remains stable.

**Figure 7:**
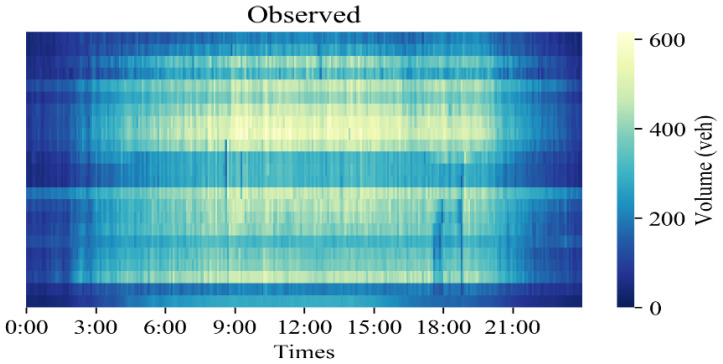
Original completed observed data for 30 June 2014 from PEMS dataset.

**Figure 8:**
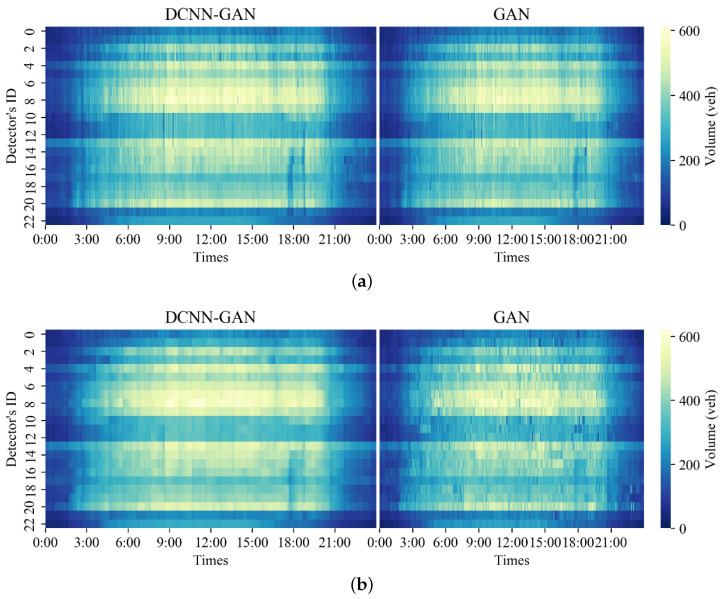

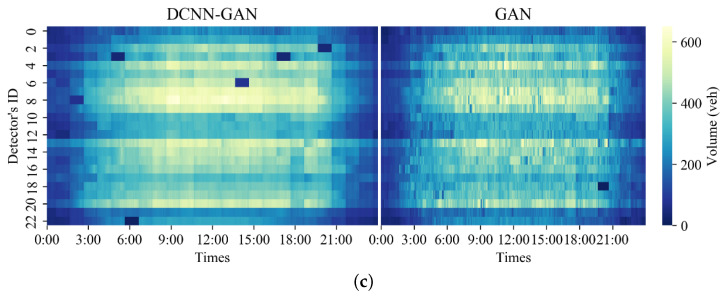
Comparison of repaired data for 30 June 2014 between DCNN-GAN and GAN under MCAR mode: (**a**) data distribution with 10% missing rate; (**b**) data distribution with 40% missing rate; (**c**) data distribution with 70% missing rate.

**Figure 9:**
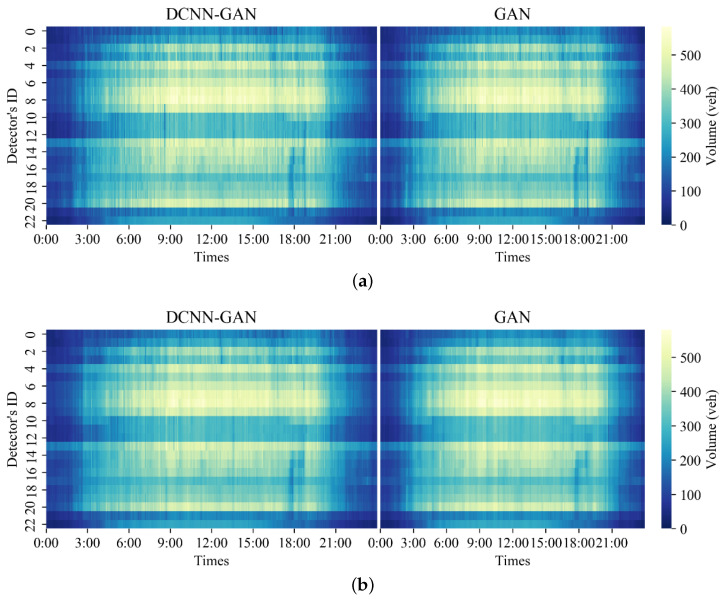

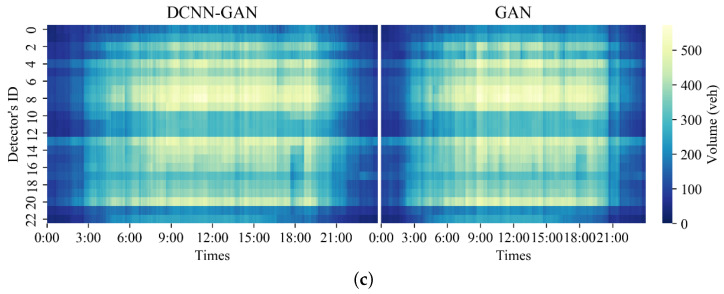
Data repair comparison for 30 June 2014 between DCNN-GAN and GAN under MCART mode: (**a**) data distribution with 10% missing rate; (**b**) data distribution with 40% missing rate; (**c**) data distribution with 70% missing rate.

In order to further compare the deduction and generation results of DCNN-GAN, the deduction and generation results of DCNN-GAN and GAN were compared, and the repair performance of DCNN-GAN was further described by comparing the thermal diagram of original data with the thermal diagrams of DCNN-GAN and GAN after data repair in the two missing data modes. Figure 7, Figure 8 and Figure 9 show the thermal diagram of the original data and the comparison of repair results between DCNN-GAN and GAN.

Figure 8 shows the repair prediction results of DCNN-GAN and GAN in MCAR deletion mode. It can be seen that as the missing rate of the traffic state data increases, the deduction results of GAN gradually encounter difficulty in characterizing the original data. On the other hand, as the deletion rate increases, DCNN-GAN remains able to stably characterize the original data distribution.

Figure 9 shows the repair results of DCNN-GAN and GAN in MCART mode. It can be seen that as the data deletion rate increases, the repair results of DCNN-GAN remain stable and continue to characterize the distribution of original data well. As the data loss rate increases, the distribution of the data repaired by GAN begins to show more serious fuzziness than with DCNN-GAN.

### 4.6. Experiment and Analysis of Seattle Dataset

In order to verify the effectiveness and applicability of the DCNN-GAN model, a comparative experimental analysis was carried out on the Seattle dataset. Similar to the PEMS dataset, the deduction generation errors of different models on the Seattle dataset are summarized in Table 3 to verify the deduction and repair performance of DCNN-GAN model. It can be seen that the prediction error of DCNN-GAN on the Seattle dataset always remains the best, and when the data loss rate is 70%, the prediction error of DCNN-GAN is always less than 8%. In MCART loss mode, the error of DCNN-GAN is always less than 7%, and the MAPE error of the other models when the loss rate is 20% exceeds the error of DCNN-GAN when the loss rate is 70%.

In addition, it can be seen that on both the PEMS and Seattle datasets, the repair errors of the models are generally higher in MCAR missing data mode than in MCART mode. This is because the spatiotemporal information in the traffic state data is more incomplete in MCAR mode, increasing the difficulty of data deduction and repair.

**Figure 10:**
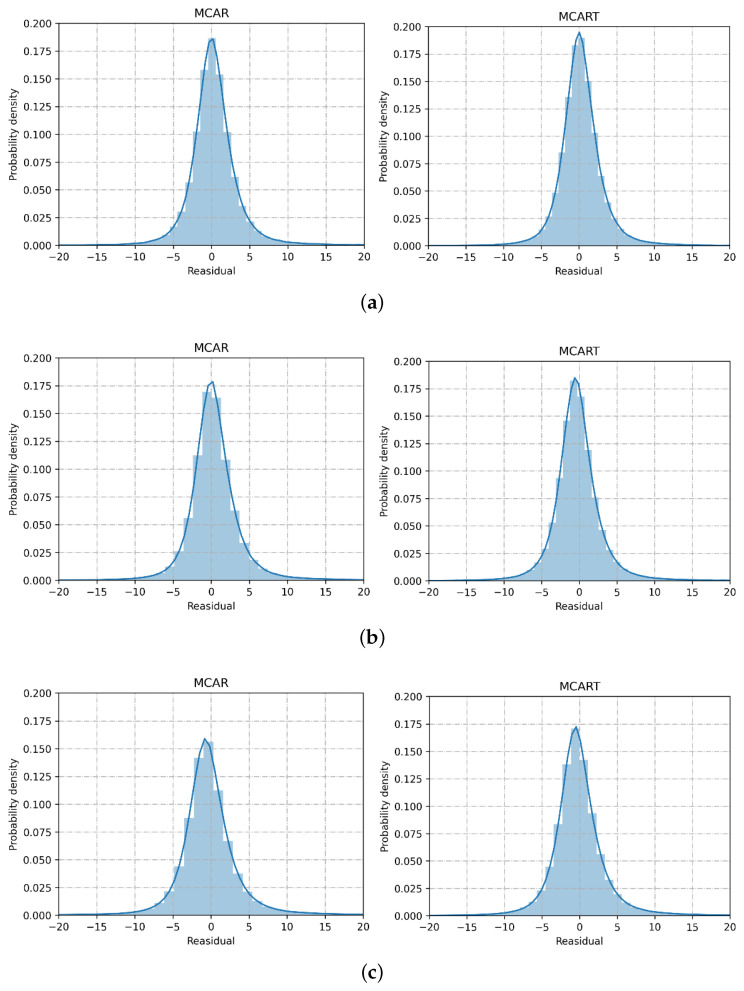
Residual distribution of the observed and imputed data with different missing data rates: (**a**) data distribution with 10% missing rate; (**b**) data distribution with 40% missing rate; (**c**) data distribution with 70% missing rate.

Figure 10 shows the residual distribution of the observed and imputed data at different loss rates. In each subfigure, the left and right figures respectively represent the residual distributions of the MCAR and MCART patterns. When the deletion rate is from 10% to 70%, the data with a residual close to zero gradually decrease. When the data deletion rate is as high as 70%, 16% of the residuals remain distributed near zero in Figure 10c. Moreover, the distribution of our model changes little with different deletion rates, indicating that the proposed model is stable. Overall, the proposed model has satisfactory performance in both MCAR and MCART modes even when the deletion rate is high.

### 4.7. Discussion

In this paper, we utilize DeepWalk to reconstruct the road network structure. We use DCNN as the internal structure of the generator for better extraction of the spatial features, and achieve effective data imputation through adversarial training using WGAN. In experiments on two datasets, we compare the performance of the model under different missing data modes and rates. The results demonstrate that DCNN-GAN can effectively perform traffic state data imputation under both MCAR and MCART missing data modes and maintain good performance even at higher missing data rates.

## 5. Conclusions

In this paper, a deduction and generation method based on DCNN-GAN for repairing missing road network data is proposed to solve the problem of detectors installed in road networks failing to capture data due to uncontrollable reasons. These data may be required for subsequent work in traffic management. The method that we propose uses a graph embedding algorithm to construct a road network structure based on spatial correlation instead of the original road network structure; through the generation of a network for confrontational training, it is possible to generate the missing data based on the known data of the road network. In the generator part of the network, the spatiotemporal features of the reconstructed road network are extracted by diffusion convolution to realize the generation of missing data. In comparison experiments conducted on two real traffic datasets, the proposed model’s data repair results are better than other comparison models.

## Figures and Tables

**Table 1:** Effect of diffusion convolution steps on the results in MCAR missing mode.

Diffusion Convolution Step Size	1	2	3	4
RMSE	25.77	25.25	25.63	25.38
MAE	19.23	19.04	19.18	19.07
MAPE	7.94	7.75	7.88	7.82

**Table 2:** Comparison of experimental results under MCAR and MCART missing data modes on PEMS-BAY dataset.

Missing Type			MCAR				MCART (%)			
**Model**			**DSAE**	**GAN**	**BGCP**	**DCNN-GAN**	**DSAE**	**GAN**	**BGCP**	**DCNN-GAN**
		RMSE	32.49	27.91	31.87	25.69	32.08	28.89	29.34	25.18
	10	MAE	24.78	20.47	22.6	19.16	24.15	20.64	21.46	18.67
		MAPE	9.65	8.15	9.02	7.81	9.21	8.68	8.66	7.39
		RMSE	34.57	28.12	35.38	26.19	33.94	29.82	29.51	25.21
	20	MAE	25.15	20.88	24.82	19.74	24.78	20.97	21.55	18.67
		MAPE	9.96	8.82	9.77	8.12	9.67	8.85	8.68	7.69
		RMSE	35.29	29.15	38.24	25.55	34.64	30.67	29.89	26.09
	30	MAE	25.62	21.53	26.59	19.16	25.13	20.14	21.68	19.41
		MAPE	10.34	8.72	10.36	8.10	10.15	8.74	8.87	7.92
		RMSE	37.28	30.31	39.35	26.34	36.67	31.78	39.57	29.17
Missing rate (%)	40	MAE	26.67	22.18	28.27	19.4	25.82	22.33	22.39	21.16
		MAPE	10.85	9.28	11.09	8.21	10.63	9.48	9.34	8.63
		RMSE	38.32	31.13	42.32	30.96	37.24	31.87	30.77	28.71
	50	MAE	27.29	20.07	30.59	21.31	26.45	22.29	22.96	21.37
		MAPE	11.24	8.43	11.89	8.74	10.96	9.44	9.75	8.79
		RMSE	39.56	32.84	45.13	31.11	38.42	32.74	31.52	30.23
	60	MAE	27.86	21.96	32.37	21.33	27.53	23.31	23.58	22.33
		MAPE	11.59	9.44	12.61	9.17	11.25	10.28	10.55	9.19
		RMSE	41.38	47.08	47.65	44.70	39.74	34.02	32.17	30.88
	70	MAE	28.43	25.93	34.12	25.74	28.19	24.04	24.27	22.85
		MAPE	12.16	11.24	13.32	10.84	11.82	11.54	10.83	9.18

**Table 3:** Comparison of experimental results under the MCAR and MCART missing data modes on the Seattle dataset.

Missing Type			MCAR				MCART (%)			
**Model**			**DSAE**	**GAN**	**BGCP**	**DCNN-GAN**	**DSAE**	**GAN**	**BGCP**	**DCNN-GAN**
		RMSE	5.09	4.28	4.70	3.32	5.72	4.15	4.76	3.12
	10	MAE	3.94	2.97	3.09	2.19	3.67	3.11	3.14	2.08
		MAPE	11.07	6.87	8.60	4.95	10.62	6.65	8.76	4.59
		RMSE	6.22	4.38	4.71	3.39	5.81	4.28	4.77	3.22
	20	MAE	4.15	3.05	3.09	2.22	3.72	2.97	3.14	2.17
		MAPE	11.46	7.09	8.61	5.12	10.7	6.58	8.71	4.75
		RMSE	6.56	4.52	4.72	3.5	5.71	4.28	4.79	3.33
	30	MAE	4.38	2.98	3.10	2.26	3.67	3.05	3.16	2.24
		MAPE	12.1	7.52	8.63	5.27	10.45	6.85	8.78	5.04
		RMSE	6.96	4.69	4.74	3.66	5.75	4.63	4.77	3.40
Missing rate (%)	40	MAE	4.71	3.07	3.11	2.33	3.69	3.04	3.15	2.27
		MAPE	12.98	7.84	8.67	5.55	10.68	7.17	8.74	5.10
		RMSE	7.33	5.02	4.74	4.03	5.77	4.74	4.85	3.64
	50	MAE	4.94	3.44	3.12	2.45	3.7	3.14	3.19	2.34
		MAPE	13.69	7.94	8.67	5.96	10.72	7.25	8.87	5.49
		RMSE	7.81	6.02	4.78	5.02	5.82	4.64	4.89	3.8
	60	MAE	5.27	3.72	3.13	2.69	3.72	3.15	3.22	2.42
		MAPE	14.7	8.50	8.74	6.52	10.79	7.71	8.93	5.76
		RMSE	8.22	7.60	4.81	6.59	5.83	4.72	4.97	3.95
	70	MAE	5.55	4.36	3.16	3.35	3.73	3.17	3.27	2.54
		MAPE	15.56	9.01	8.82	7.96	10.76	8.34	9.08	6.11

## Data Availability

Data are contained within the article.
